# Tau and β-Amyloid Relevant Pathology as a Central Therapeutic Target in Alzheimer’s Disease

**DOI:** 10.3390/biom16040595

**Published:** 2026-04-17

**Authors:** Lidia Strużyńska, Kamil Adamiak, Marta Sidoryk-Węgrzynowicz

**Affiliations:** 1 Laboratory of Pathoneurochemistry, Department of Neurochemistry, Mossakowski Medical Research Institute, Polish Academy of Sciences, 5 Pawińskiego Str., 02-106 Warsaw, Poland; lidkas@imdik.pan.pl (L.S.); kadamiak@imdik.pan.pl (K.A.); 2 Doctoral School of Translational Medicine, Centre of Postgraduate Medical Education, 99/103 Marymoncka Str., 01-813 Warsaw, Poland

**Keywords:** neurodegeneration, amyloid β, tau, protein aggregation, drug therapy, active immunization, passive immunization

## Abstract

Alzheimer’s disease (AD) is the leading cause of dementia, responsible for approximately 60–70% of cases globally. AD is a gradually progressive neurodegenerative disorder that is characterized by widespread deposition of β-amyloid (Aβ) plaques, followed by aggregation of tau protein in the neocortex, neurodegeneration, and cognitive decline. Within these complex pathological interactions, Aβ and tau proteins, together with astrogliosis, neuroinflammation, and other factors, play a key role in the development of clinical AD. Accumulating evidence indicates that the formation of protein oligomers, followed by their aggregation into pathological fibrils, constitutes an early and critical step in the pathogenesis of the disease. Specific pathological proteins are often treated as biomarkers of particular diseases because their presence, concentration, or altered structure reflects an underlying disease process. It is well established that the Aβ and tau proteins are the key hallmarks of AD, and their mutual interaction may significantly influence the pathology of the disease. Early diagnosis is crucial for maximizing the therapeutic benefits of currently available symptomatic treatments, which can alleviate symptoms and modestly delay clinical deterioration in patients with AD. This review highlights the mechanisms involved in protein-dependent neurodegeneration and describes both traditional and novel approaches for the cure of AD. The most important aspect of this publication is the integration of the two key proteins: Aβ and tau, and the resulting shift toward a new therapeutic approach.

## 1. Introduction

Neurodegenerative disorders such as Alzheimer’s disease (AD) or frontotemporal dementia pose a significant socio-economic challenge due to their high prevalence and, at the same time, ineffective prevention and treatment. They are often called proteinopathies due to the presence of neurotoxic forms of specific proteins that lose their physiological roles [1,2,3]. Proper folding of newly synthesized polypeptide chains into their native conformations is critical for attaining a functional protein state. Conversely, partial or complete unfolding can induce misfolding, enabling monomeric proteins to aggregate into structurally ordered assemblies. Notably, amyloid aggregates exhibit the polypeptide chains forming the cross-β structure located perpendicularly to the fiber axis, a defining feature of protein aggregation in diseases such as AD. Soluble aggregates arising during protein aggregation, known as oligomers, are highly heterogeneous and can undergo rapid conversion into larger, toxic fibrillar structures (protofibrils) within the diseased brain, as observed in AD [4]. Disease-specific misfolded protein/s found in the AD brain include beta-amyloid (Aβ) and hyperphosphorylated tau protein. It is well established that Aβ (https://www.sciencedirect.com/topics/biochemistry-genetics-and-molecular-biology/amyloid-beta, accessed on 1 April 2026) and tau proteins (https://www.sciencedirect.com/topics/neuroscience/tau-protein, on 1 April 2026) misfold, self-assemble, and propagate by an endogenous mechanism that closely resembles the seeded aggregation and spreading of prion protein (PrP) (https://www.sciencedirect.com/topics/pharmacology-toxicology-and-pharmaceutical-science/prion-protein, accessed on 1 April 2026) in Creutzfeldt–Jakob disease and other prionopathies [5]. Aβ plaques disrupt neural circuits and damage neighboring brain cells, while smaller, soluble Aβ oligomers, representing the most toxic formations, interfere with synaptic function, impair neuronal signaling and plasticity, and disrupt the normal function of neurons and glial cells. In parallel, mutations in tau give rise to a group of neurodegenerative disorders collectively known as primary tauopathies, which are characterized by distinct fibrillar structures that differ from the fold observed in the paired-helical filaments typical of AD. Protein assembly begins in specific regions of the brain, depending on the type of neurodegenerative disease, and then spreads to other areas as the disease progresses [6]. Impaired clearance by the autophagic–lysosomal network is a major contributor to the accumulation and propagation of oligomeric neurotoxic proteins. Moreover, other proteostatic pathways are disrupted in neurodegenerative diseases, including chaperone-mediated autophagy, the ubiquitin–proteasome system, extracellular proteolytic degradation, and transport across the blood–brain barrier into the circulation [7]. Despite the development of new diagnostic methods, AD is often diagnosed too late, in the second or even third stage of the disease. The diagnosis of AD comprises multiple components, including epidemiological, neuropsychological, electroencephalographic, and magnetoencephalographic assessments, as well as neuroimaging techniques [8].

Brain imaging and an assessment of neuronal metabolic changes include the use of single-photon emission-computed tomography (SPECT), which allows for the estimation of cerebral blood flow; positron emission tomography (PET); and magnetic resonance imaging (MRI). Amyloid-Related Imaging Abnormalities (ARIA) is a term used to describe abnormalities visible on brain imaging that may occur in patients treated with anti-amyloid drugs (e.g., modern therapies). These methods are used to identify brain dysfunctions and differential diagnosis, allowing the determination of the extent and topography of neuronal degeneration, as well as the exclusion of tumors or hematomas and, consequently, vascular dementia [9]. These days, ARIA is most prominently linked to the side effects of passive immunotherapies with monoclonal antibodies. In cases of familial occurrence of the disease, additional diagnostic procedures may be performed, including molecular analysis of the presenilin 1 (PSEN1) and presenilin 2 (PSEN2) genes. Mutations in these genes (e.g., p.A136V) have been identified in patients with early-onset Alzheimer’s disease [9]. According to the widely accepted hypothesis that β-amyloid accumulation in the brain initiates the pathophysiology of Alzheimer’s disease, early disease stages can be diagnosed through positron emission tomography (PET) imaging or by measuring decreased concentrations of amyloid peptides in cerebrospinal fluid [10]. Early diagnosis is essential for maximizing the therapeutic efficacy of currently available symptomatic treatments, which can alleviate symptoms and delay clinical progression in patients with AD. This review focuses on describing the most common specific proteins associated with neurodegenerative processes related to AD and presents old and novel approaches targeting these pathological proteins. Despite decades of research, effective therapy for AD remains insufficient, and this review aims to critically evaluate the current attempts at targeted intervention against key proteins that have progressed to clinical trials. A search of databases, such as PubMed, Scopus, or the U.S. Food and Drug Administration (FDA) archive, was conducted to extract relevant data. Keywords for database searches included the following: Alzheimer’s disease, neurodegeneration, tau, beta-amyloid, traditional therapy, monoclonal antibodies, and passive and active immunization. Experimental and review articles on AD in various cell types and in vivo models were included.

## 2. Alzheimer’s Disease

### 2.1. AD Background

The most common group of amyloid-derived diseases in the central nervous system (CNS) is β-amyloidoses, in which the main component of amyloid is amyloid-β (Aβ) [11]. Aβ peptides form amyloid deposits in blood vessels exhibiting features of congophilic angiopathy, as well as interstitial deposits in the form of senile plaques, observed both in physiological aging and in Alzheimer’s disease and Down syndrome [12]. The presence of amyloid beta deposits in other neurodegenerative diseases can be attributed to mixed pathologies that are found in a substantial fraction of patients.

AD is the most widespread type of dementia and one of the most common chronic diseases, affecting almost 50 million elderly individuals worldwide. One of the most common dementing disorders in humans is AD. There are currently over 44 million people living with dementia worldwide, and this number is predicted to more than triple by 2050 [13,14]. Most studies estimate the prevalence of AD to be between 1.9% and 5.8% in the general population aged 65 years and older. Age-specific studies of the prevalence of dementia have demonstrated that until the age of 74, the incidence rate remains below 5%, while by the age of 85, it increases to 20–30%. In addition to age, family history represents an important risk factor for primary degenerative dementia. The risk of developing the disease among first-degree relatives is approximately 19%, while among second-degree relatives, it is about half that value, reaching approximately 10% [15]. AD manifests as progressive impairments in memory and cognitive function. At the cellular level, it is defined by neuronal and synaptic loss, vascular and interstitial β-amyloid deposits forming congophilic angiopathy and senile plaques, and neurofibrillary degeneration within affected neurons. In 1987, it was demonstrated that the Aβ peptide is the product of proteolytic processing of a larger precursor protein known as AβPP [16]. This protein is a glycoprotein with a molecular weight of 100–140 kDa, encoded by a gene located on the long arm of chromosome 21. As a result of alternative mRNA splicing, at least ten AβPP isoforms of various lengths, ranging from 365 to 770 amino acids, may be generated. AβPP is a transmembrane protein consisting of an extracellular N-terminal domain, a transmembrane domain, and a short C-terminal domain located in the cytosol. The N-terminal fragment of AβPP, released into the extracellular space as a result of proteolytic cleavage, contains sites for glycosylation, sulfation, and binding of proteoglycans and heparin [17]. Aβ peptides constitute a small fragment of the precursor protein, located in the extracellular and transmembrane domains. They typically consist of 39–43 amino acids. It is widely accepted that there are two main catabolic pathways for AβPP metabolism: the secretory pathway and the endosomal–lysosomal pathway [18]. The latter leads to the formation of amyloidogenic Aβ peptides. The key enzymes involved in this process are β- and γ-secretases [19]. According to current evidence, the diffuse plaques observed during physiological aging are mainly composed of the (Aβ17-42) peptide, generated via the secretory pathway from AβPP. Aβ1-42 peptides constitute the main components of diffuse plaques and early senile plaques in AD, whereas Aβ1-40 peptides appear in later stages and represent one of the main components of mature plaques [20]. In general, Aβ1-42 is more aggregation-prone and strongly linked to plaque formation, while Aβ1-40 is more abundant but less pathogenic. The ratio between them is an important indicator used in Alzheimer’s diagnostics and research. Studies show that the Aβ42/Aβ40 ratio correlates better with amyloid PET imaging, reduces false-positive and false-negative results, and improves differentiation between AD and other dementias. The Aβ42/Aβ40 ratio in blood is emerging as one of the most promising non-invasive indicators for early AD detection [21].

Previous studies have identified several structural domains within the Aβ peptide that are responsible for its biochemical interactions with proteins and other cellular components. The N-terminal domain of Aβ is involved in peptide adhesion processes, activation of the complement system component C1q, and binding of α-antichymotrypsin [22]. The N-terminal domain is also responsible for binding to tau protein [23]. In addition to the presence of interstitial and vascular Aβ deposits and the loss of neurons and synapses, neurofibrillary degeneration is an important component of AD pathology. Neuropathological studies have demonstrated that early neurofibrillary changes may occur during normal aging, whereas in AD, they initially appear in specific brain structures and progressively involve other regions [24].

Mutations in the *APP* gene (located on chromosome 21) shift processing toward the amyloidogenic pathway, leading to the elevated production of Aβ42 (toxic form) and the formation of amyloid plaques in the brain and an earlier onset of disease. Common *APP* mutations include an increasing total Aβ production Swedish mutation (KM670/671NL), increasing the proportion of Aβ42 London mutation (V717I), and leading to the aggregation of the Aβ Arctic mutation (E693G) [25,26,27]. A recent study revealed a novel missense mutation in *APP* [28], E674Q, in an index patient with late-onset familial AD. This mutation is potentially disease-causing by facilitating the beta-site APP cleaving enzyme 1-mediated processing of APP and the production of Aβ [28].

Rare cases of early-onset familial AD are caused by mutations in the genes encoding amyloid precursor proteins, namely, PSEN1 and PSEN2 [5]. One of the most significant genetic risk factors for AD is the apolipoprotein E (ApoE) gene, located on chromosome 19 [29]. Apolipoprotein E, encoded by this gene, is a serum protein involved in cholesterol transport. Three ApoE isoforms have been identified: ApoE2, ApoE3, and ApoE4, each encoded by a corresponding allele. Particular importance is attributed to the ApoE4 allele, which is present in approximately 52% of individuals suffering from AD, compared with only about 16% in control populations. Homozygous carriers of the ApoE4 allele exhibit a substantially higher risk of developing the disease compared with individuals carrying a single copy of this allele. In general, the ApoE3 allele is risk-neutral, the ApoE4 allele increases the risk of developing AD, and the ApoE2 allele is neuroprotective, contrasting with the ApoE4 relevant effects [30].

In addition to the presence of interstitial and vascular Aβ deposits and the loss of neurons and synapses, neurofibrillary degeneration is an important component of AD pathology. Neuropathological studies have demonstrated that early neurofibrillary changes may occur during normal aging, whereas in AD, they initially appear in specific brain structures and progressively involve other regions [24].

**Figure 1:**
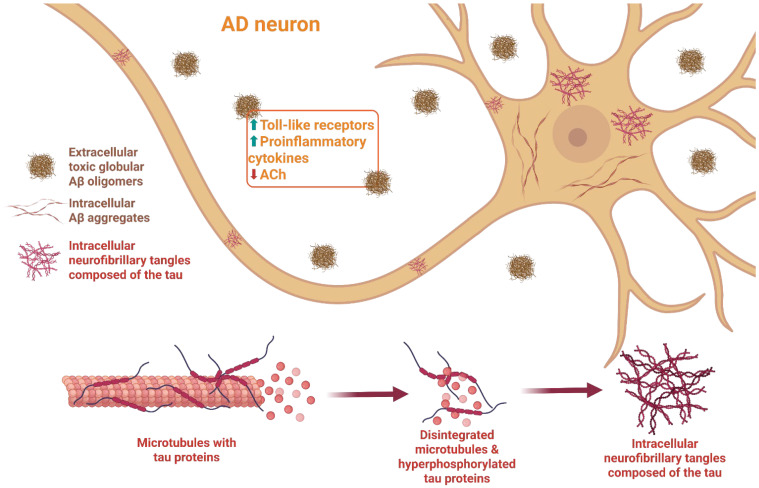
β-amyloid and tau pathology in AD. In AD, the amyloid precursor protein is processed into Aβ peptides that accumulate, form plaques, and exert detrimental effects on neuronal cells. In AD, brain neurofibrillary tangles (NFTs) are located inside neurons and spread along neuronal cells. In addition, increased levels of dendritic tau cause neurons to be vulnerable to pathological Aβ. The synergistic interaction between Aβ and tau at the synapse contributes to synaptic dysfunction, loss, and neurodegeneration. Created in BioRender. Adamiak, K. (2026) https://BioRender.com/hh5o9w2 (accessed on 10 April 2026).

**Figure 2:**
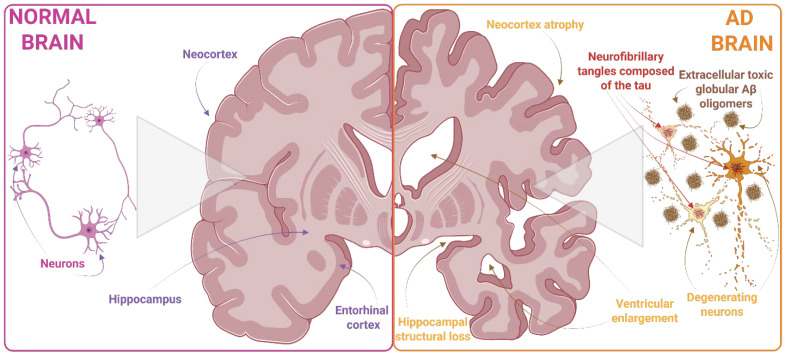
Propagation of β-amyloid tau pathology in AD brain. Amyloid precursor protein is processed into amyloid Aβ peptides, leading to the formation of extracellular amyloid plaques. The tau pathology in the AD brain’s neurofibrillary tangles is located inside neurons. These spread along a neural network in a stereotypical manner in cells and involve micropinocytosis and related mechanisms. In addition, an increasing amount of dendritic tau makes neurons vulnerable to pathological Aβ. Created in BioRender. Adamiak, K. (2026) https://BioRender.com/csagngu (accessed on 10 April 2026).

Extracellular Aβ plaques and intracellular neurofibrillary tangles of hyperphosphorylated tau constitute the two principal pathological hallmarks of AD and serve as widely accepted targets for its diagnosis [Figure 1 and Figure 2].

### 2.2. AD as a Tauopathy Disease

Depending on the underlying cause(s), tauopathies are classified as primary and secondary, where tau protein abnormalities are the defining etiological feature of the disease or are a consequence of other disease processes, respectively. In primary tauopathies, which include frontotemporal dementia (FTD), progressive supranuclear palsy (PSP), corticobasal degeneration (CBD), and Pick’s disease, pathological tau aggregates are the primary cause of subsequent neurodegeneration. In secondary tauopathies, certain other factor(s) may lead to tau aggregation.

AD is the most common secondary tauopathy, in which abnormally hyperphosphorylated tau protein forms fibrillar aggregates, known as neurofibrillary tangles (NFTs), that play a key role in neurodegeneration. Increased levels of both total and phosphorylated tau protein in cerebrospinal fluid and plasma are considered key indicators of disease progression [31] [Figure 1].

Tau is a highly expressed soluble phosphoprotein present in neurons of both the central and peripheral nervous systems [32]. Typically, tau is predominantly enriched in axons, where it interacts with microtubules (MTs). It plays a fundamental role in neuronal function by facilitating the assembly and stabilization of MTs, supporting neurodevelopment, and maintaining axonal integrity and intracellular transport [33,34]. The human *MAPT* gene, which encodes tau, consists of 16 exons, 11 of which are expressed. In the adult human brain, tau exists in six major isoforms generated by alternative splicing of mRNA involving exons 2, 3, and 10. Notably, alternative splicing of exon 10 regulates the production of tau isoforms containing three (3R) or four (4R) microtubule-binding repeats, influencing tau’s functional properties and its propensity for aggregation. Alternative splicing of exon 10 affects the synthesis of tau protein, whose C-terminal region contains either three (3R) or four (4R) microtubule-binding repeat motifs. Under physiological conditions in the adult human brain, the 3R:4R isoform ratio is approximately 1:1 [35]. The length of the synthesized tau isoforms ranges from 352 to 441 amino acids. In pathological conditions, this isoform ratio becomes disrupted, leading to an excessive accumulation of specific tau isoforms, which subsequently leads to abnormal phosphorylation and impaired binding of tau protein to microtubules.

Loss of tau’s ability to bind MTs leads to cytoskeletal destabilization, which results in neurodegeneration and neuronal death. Pathological aggregation of tau into hyperphosphorylated filaments drives its conversion into paired-helical filament (PHF) structures. This process leads to microtubule depolymerization and tau dissociation, ultimately resulting in the formation of more complex neurofibrillary tangles (NFTs). NFTs composed of hyperphosphorylated tau aggregates are observed in both inherited and sporadic dementias, including progressive supranuclear palsy, corticobasal degeneration, frontotemporal dementia, and Parkinsonism linked to chromosome 17 (FTDP-17), and other tauopathies, including AD [3,36]. Within Alzheimer’s disease, neurofibrillary tangles (NFTs) spread in a stereotyped manner through defined brain regions, and their distribution and density form the basis of the Braak stage, which is widely used to evaluate disease progression [24,37] [Figure 2]. The process of NFTs formation can be initiated by several factors, including genetic factors, oxidative stress, inflammation in the CNS, toxins, or disturbed carbohydrate–lipid metabolism. Importantly, as demonstrated in animal models, reducing tau protein expression can prevent behavioral and cognitive changes, showing that tauopathies are closely correlated with dementia.

According to the hypothesis linking the pathogenesis of AD with tau protein, a key element of the neurodegenerative process is tau hyperphosphorylation, which leads to the formation of NFTs [38,39]. Therefore, reducing NFT-induced neurodegeneration may represent a potential therapeutic target in AD and other tauopathies [40].

## 3. Therapeutic Strategies in Alzheimer’s Disease

### 3.1. “Traditional” Therapies

Despite extensive research over the past several decades, Alzheimer’s disease remains the most common and currently incurable form of dementia. The earliest “traditional” therapeutic approaches focused on the inflammatory component that accompanies neurodegenerative processes and has been demonstrated to play a role in the development of AD. For example, complement component C1q has been reported to enhance Aβ deposition in fetal hippocampal cells [41]. Postmortem studies of individuals suffering from AD have revealed the presence of numerous inflammatory mediators, such as acute-phase proteins, including α2-macroglobulin, cytokines of the TNF family, interleukin-1β and IL-6, integrins, and complement components C1q, C4, and C3 [42]. It has been shown that the use of nonsteroidal anti-inflammatory drugs (NSAIDs) in patients with rheumatoid diseases reduces the risk of AD, and that the nonspecific anti-inflammatory treatment in AD patients may alleviate the severity of clinical symptoms to some extent. Considerable hope was placed in research on trophic factors (NGF, BDNF, CNTF), which are essential for maintaining neuronal function [43]. However, clinical trials did not yield positive results, as growth factors generally do not cross the blood–brain barrier (BBB).

The use of drugs that enhance the activity of the neurotransmitter system represents another pharmacological attempt to counteract the disease. Cholinesterase inhibitors are the main and most established “traditional” pharmacological strategy for symptomatic treatment of cognitive decline in AD and some other dementias. Cholinesterase inhibitors block the enzyme acetyl cholinesterase, which normally breaks down acetylcholine [44]. The inhibition of this enzyme by drugs (e.g., donepezil, rivastigmine, galantamine) increase the amount and duration of acetylcholine in synapses, improving communication between nerve cells. These medications are typically used for mild to moderate Alzheimer’s disease, although donepezil is also approved for severe stages. The oldest agent within this category, physostigmine, functions as a non-selective inhibitor of cholinesterase enzymes, affecting both central and peripheral nervous system targets [45]. The first drug approved in the United States for the treatment of AD was Cognex (tacrine), an irreversible inhibitor of acetylcholinesterase in the CNS, which additionally acts on cholinergic receptors and enhances the release of neurotransmitters, including acetylcholine. However, this drug produces numerous adverse side effects, such as nausea, vomiting, and psychomotor agitation, as well as decreased arterial blood pressure. Another acetylcholinesterase inhibitor, rivastigmine, exhibits high affinity for the enzyme in the hippocampus and cerebral cortex [46]. However, the strong potency of rivastigmine, resulting from its dual inhibitory mechanism, has been postulated to cause increased nausea and vomiting during the titration phase of oral treatment [47]. The most common clinical positive effects following drug acting as acetyl cholinesterase inhibitors are memory and cognition, attention and thinking, functioning, and, rarely, behavioral symptoms. However, they do not stop or reverse disease progression—they mainly slow symptomatic decline for a period of time but do not modify the underlying disease process.

Among the drugs that affect cerebral blood flow, agents with vasodilatory effects are used, including calcium channel blockers. One example is cinnarizine, which also exhibits antihistaminic and anti-serotonergic activity. Flunarizine shows similar properties. The use of vascular drugs in AD is marginal and usually supportive, limited mainly to situations of coexisting cerebral perfusion disorders.

Psychiatric symptoms common in the disease, such as anxiety and depression, are treated with psychotropic drugs. Due to their different receptor binding profiles, atypical antipsychotic drugs are associated with a lower risk of inducing extrapyramidal symptoms, such as muscle rigidity, bradykinesia, tremor, and gait disturbances. The most commonly prescribed substances from this group include clozapine, risperidone, and olanzapine, while among the first-generation antipsychotics, haloperidol is the most frequently used [48]. Among the antipsychotic drugs officially approved in Europe for the treatment of behavioral disorders and persistent aggression in patients with moderate to severe AD-related dementia is risperidone [49]. It is worth noting that the use of psychotropic drugs significantly complicates the course of the disease by negatively affecting cognitive processes [46].

Memantine, patented in 1966, is a medication commonly used to treat moderate to severe forms of AD. Memantine targets the NMDA (N-methyl-D-aspartate) receptors, which are activated by the neurotransmitter glutamate. It is well established that in AD pathology, excess glutamate overstimulates NMDA receptors, leading to excitotoxicity and damage or death of neurons, the processes responsible for cognitive decline [50]. Memantine partially blocks these receptors, which reduces harmful overstimulation, protects neurons from glutamate toxicity, maintains normal cellular metabolism, and helps maintain cognitive function. Therefore, memantine is considered a fundamental therapy for moderate to severe AD, because of the beneficial effects (e.g., improvement or stabilization of memory, maintaining daily functioning, reduction of behavioral symptoms). Common side effects of memantine use include dizziness, headache, confusion, and constipation [51]. To overcome these challenges and given the clinical heterogeneity of AD, the most effective therapeutic approach may involve combination therapy using multiple drugs rather than relying on a single agent [52].

In the progression of AD, the pathology of Aβ and tau disrupts mitochondrial mass, mitochondrial dynamics, transport, and morphology. Imbalances in mitochondrial fission and fusion have been noticed in several brain regions of AD [51]. Mitochondrial dysfunction and oxidative stress contribute to neuronal damage in AD by reducing energy production and increasing harmful reactive oxygen species, accelerating neurodegeneration. In AD, mitochondria become damaged or function less efficiently, leading to increased production of reactive oxygen species (ROS) and reduced neuronal energy output. Oxidative stress arises when ROS generation exceeds the brain’s antioxidant defenses, resulting in lipid peroxidation (damage to cell membranes), protein oxidation, and damage to both nuclear and mitochondrial DNA [53]. These effects contribute to neuronal injury and death, and accelerate neurodegeneration [54]. Strategies aimed at preserving mitochondrial function and enhancing antioxidant defenses are actively being investigated as potential therapeutic approaches for AD [55].

As can be seen, pharmacological approaches applied over the past several decades have not resulted in a significant breakthrough in the treatment of dementia. Nevertheless, modern research tools and current research directions appear much more promising in the therapy of neurodegenerative diseases. Alzheimer’s disease is thought to progress through two stages. The first stage is marked by the emergence and gradual spread of aberrant Aβ and associated pathological processes. The second stage involves a complex cascade of secondary changes, including tau-containing neurofibrillary tangles, neuroinflammation, vascular abnormalities, and widespread neurodegeneration. During this stage, disease progression appears to be at least partially independent of Aβ deposition [56]. The pathogenic properties of Aβ make it an especially attractive target for early preventive interventions, although tauopathy, as a defining pathological feature of AD, has also been explored as a target in immunization strategies. This bi-phasic strategy of AD pathogenesis has important implications for both treatment and prevention strategies [57].

### 3.2. Aβ-Targeted Therapies

#### 3.2.1. Active Immunization

In active immunization, the administration of Aβ or its fragments stimulates the immune system, thereby triggering an immune response by activating the phagocytic capacity of microglia, which leads to the production of endogenous antibodies against Aβ [58,59]. This type of immunotherapy for AD began in earnest in 1999, when researchers first conducted active immunization of transgenic mice that had been genetically modified to overproduce AβPP. In the experiment, synthetic Aβ polymers were used, which are capable of eliciting an immune response. The results were promising. A significant reduction in Aβ accumulation in the animal brain was observed, providing the first evidence that immune stimulation can effectively reduce the pathological plaques characteristic of AD [60,61,62,63,64,65,66]. This discovery generated significant interest in the research into the use of immune mechanisms for the treatment and prevention of AD [Table 1].

The next stage was the development of the AN1792 vaccine in 2001, directed against full-length Aβ, which entered clinical trials [67]. The study was stopped in phase II due to the occurrence of T-cell-induced meningoencephalitis. To limit the excessive immune response, a second generation of vaccines lacking T-cell epitopes was developed. An example is CAD106, which is currently in phase II/III clinical trials. The advantages of active immunotherapy include low financial costs and a short time of drug administration, and the resulting antibodies persist in the body for a long time [68]. On the other hand, its disadvantages include immune responses and side effects that are difficult to precise, especially in elderly individuals.

**Table 1:** Preclinical studies of Aβ immunotherapy in mouse models of AD, for example, of active and passive approaches.

Mouse Model	Immunization	Key Effects	References
PDAPP	**Active immunization** 3D6 directed against Aβ(1-5); 12B4 directed against Aβ(3-7)	Aβ reduction by active immunization protects against the progressive loss of synaptophysin	[69]
PDAPP	**Passive immunization** Aβ1–42; Aβ1–5; Aβ3–7	Anti-Aβ (3D6, 12B4) hinders neuritic degeneration and synaptic pathology in PDAPP mice	[69]
APP + PS1	Aβ1–42	Cognitive improvement	[65]
Tg2576	Aβ25–35	Time course of plaque reduction, behavioral improvement	[64]
Tg2576	Aβ1–16	Rapid reduction of diffuse deposits; decline of neuritic plaques via microglial activation	[63]
PDAPP	266	Reduced plaque burden	[61]
PDAPP	3D6 (Aβ1–5), 10D5 (Aβ3–6)	Presence of antibodies bound to amyloid plaques; clearance of plaques	[60]
PDAPP, Tg2576	3D6, 10D5	Clearance of diffuse Aβ deposits	[62]

#### 3.2.2. Passive Immunization

Passive immunization with humanized anti-Aβ antibodies has emerged as a major strategy in the development of therapeutics for Alzheimer’s disease. In this approach, externally produced antibodies are administered via intravenous infusion or subcutaneous injection. For example, bapineuzumab represents a humanized version of a murine antibody that was initially evaluated in preclinical studies. Monoclonal antibodies (mAbs) can also be fully human, an example of which is gantenerumab. Such antibodies are produced in transgenic mice that have been genetically modified to carry a human immunoglobulin locus, whereas humanized mAbs are initially generated in wild-type mice with an endogenous murine immunoglobulin locus. Compared to humanized murine mAbs, fully human mAbs are considered safer and more effective due to a lower incidence of adverse effects.

The antibodies that have advanced farthest in clinical development for Alzheimer’s disease include bapineuzumab, solanezumab, crenezumab, gantenerumab, aducanumab, lecanemab, and donanemab [70,71,72,73,74]. These monoclonal antibodies are designed to target different forms of Aβ, a protein that aggregates into plaques in the brains of AD patients, which are thought to contribute to neuronal damage and cognitive decline. Early-generation antibodies, such as bapineuzumab and solanezumab, were primarily aimed at clearing existing amyloid plaques or neutralizing soluble forms of Aβ, and their clinical efficacy was limited.

One of the most significant medical achievements in recent years was the introduction of two new drugs for the treatment of AD to the pharmaceutical market, approved by the FDA in June 2021. A later-generation antibody, such as aducanumab, is the first drug shown to be effective in reducing the accumulation of Aβ plaques in the brain, which are considered one of the main causes of the disease. Aducanumab is characterized by a long half-life and is administered once every four weeks. Later-generation antibodies, such as aducanumab, represent the first drugs demonstrated to effectively reduce Aβ plaque accumulation in the brain, which is considered a key contributor to the pathogenesis of Alzheimer’s disease. Aducanumab was approved by the FDA in 2021 under the accelerated approval pathway based on clinical trial data demonstrating its effect on reducing Aβ plaques. It was concluded that the drug is likely to provide clinical benefits, including improvement in the clinical condition of patients [75]. Aducanumab has been taken off the market due to a lack of efficacy at the beginning of 2024 [76]. Patients with early symptomatic AD, mild cognitive impairment, or the mild dementia stage of the disease may be eligible to switch to the donanemab and lecanemab treatment as an alternative therapy [Table 2].

Another drug approved for the treatment of AD is lecanemab, which received FDA approval in January 2023. Like aducanumab, lecanemab is a monoclonal antibody designed to reduce the accumulation of Aβ plaques in the brain. This drug was also approved under the accelerated approval pathway, which has raised controversy among some experts [82]. The phase III clinical trial successfully met its primary endpoint, demonstrating a statistically significant slowing of disease progression compared with the placebo group. After 18 months of therapy, during which patients received intravenous infusions of lecanemab every two weeks at a dose of 10 mg/kg, the rate of cognitive decline was reduced, and this improvement was accompanied by a corresponding decrease in cerebral Aβ levels. According to the results of the clinical trial, lecanemab slowed disease progression by 27% compared to placebo. However, it should be emphasized that despite the observed slowing of cognitive and functional decline in patients with early-stage AD, adverse events were also reported during treatment. Therefore, further studies are needed to more precisely assess the efficacy and safety of lecanemab in the treatment of early-stage AD. An example of unsuccessful attempts to introduce drugs to the market, as occurred in the case of aducanumab, demonstrates and confirms the complexity of treating Alzheimer’s disease and the need to further advance knowledge in the neurodegeneration area of research.

#### 3.2.3. Key Criteria for Effective Aβ Immunotherapy in Ad

Based on recent clinical trial results, the most consistent conclusion is that reducing cerebral Aβ burden can slow the progression of Alzheimer’s disease. Solanezumab selectively binds soluble Aβ, facilitating its clearance from the brain and representing a targeted approach within the passive immunotherapy strategy for AD. Although meta-analyses suggest that solanezumab may confer modest clinical benefits, large-scale clinical trials have consistently failed to meet their primary endpoints. Monomeric Aβ is highly abundant in the brain, and its complete neutralization would require stoichiometric amounts of high-affinity antibodies capable of competing with the binding of monomers to pre-existing Aβ aggregates. Importantly, extensive preclinical evidence indicates that Aβ toxicity is primarily related to its aggregated forms rather than monomeric forms, and the antibodies that demonstrated the strongest clinical signals have also caused the most pronounced reduction in aggregated Aβ. For these reasons, antibodies that broadly recognize monomeric Aβ are unlikely to represent the most effective immunotherapeutic strategy.

#### 3.2.4. Major Limitations of Therapeutic Antibodies Approach

Therapeutic antibodies such as lecanemab, donanemab, or aducanumab are large immunoglobulin G (IgG) molecules (~150 kDa), which normally cross the BBB only very poorly. Pharmacokinetic studies show that typically only about 0.1–0.3% of circulating IgG reaches the brain parenchyma [83]. Despite this very small fraction, measurable concentrations still appear in the brain interstitial fluid and cerebrospinal fluid. Anti-amyloid Aβ antibodies bind strongly to aggregates and plaques. Even low concentrations may be sufficient to occupy an accessible amyloid epitope. Once bound to amyloid plaques, IgG antibodies can engage Fc receptors on microglia, stimulating phagocytosis and clearance mechanisms that allow these antibodies to exert measurable effects. Nowadays, the study is focused on actively developing next-generation strategies to improve antibody delivery across the BBB. One promising strategy is to engineer more specific antibodies that bind both the therapeutic target and a receptor involved in transport across the BBB. Another strategy is to reduce the molecular size by using fragments such as single-chain variable fragments (scFv) or nanobodies (single-domain antibody) because these small molecules may diffuse more easily through the BBB and into brain tissue [84,85]. Although this approach is not currently on the market for AD, some preclinical and early clinical evidence indicate potential as a novel therapeutic [86].

### 3.3. Tau-Targeted Therapies in AD

Although extensive research has been done, Aβ-targeted therapies have not proven very effective in human clinical trials. Therefore, considering the recent progress in tau research, it seems that the strategy targeting tau protein may be a promising therapeutic approach in AD [40].

As an alternative to anti-Aβ therapy, tau-targeted treatment aims to inhibit tau phosphorylation and aggregation. To reduce the amount of NFTs, studies were conducted using methylene blue and, subsequently, its derivative LMTM (leuco-methylthioninium bis(hydromethanesulfonate). In murine models, these compounds have been shown to be effective as they reduced NFT-induced neuronal apoptosis. However, clinical trials have not confirmed this effectiveness [87].

Furthermore, microtubule-stabilizing agents are being investigated as a potential disease-modifying strategy in AD. In this context, the tubulin-binding, third-generation synthetic taxane TPI-287 was tested. However, clinical trials already in phase I showed that this compound was ineffective and poorly tolerated. Strategies targeting tau protein include inhibition of tau hyperphosphorylation and aggregation, as well as both active and passive immunotherapy. Inhibition of glycogen synthase kinase-3 (GSK-3) is an important therapeutic target in the treatment of neurodegenerative disorders, including AD. GSK-3 is a protein kinase involved in tau phosphorylation; therefore, its inhibition blocks the formation of NFTs. Studies have also shown the involvement of this enzyme in APP processing and Aβ production, as well as in apoptotic cell death. Among GSK-3 inhibitors, only tideglusib has progressed to phase II clinical trials for the treatment of Alzheimer’s disease. Trials in patients with mild to moderate AD demonstrated that tideglusib was generally well tolerated, but no significant clinical improvement was observed [39].

#### 3.3.1. Active Immunization

The first vaccine tested against tau protein, AADvac1, contains a synthetic peptide corresponding to the naturally occurring, truncated, and misfolded form of tau. Studies conducted in transgenic mice demonstrated that this vaccine not only reduces tau protein hyperphosphorylation and improves memory but also exhibits a favorable safety profile and induces high levels of anti-tau antibodies [38]. Subsequent studies involving patients with moderate AD showed the development of antibodies against tau protein, and the treatment was well tolerated. The results of these studies suggest that AADvac1 may slow disease progression [88].

#### 3.3.2. Monoclonal Antibodies

The tau spreading hypothesis provides a rationale for passive immunization with anti-tau monoclonal antibodies, aiming to block the seeding and propagation of extracellular tau aggregates as a disease-modifying strategy for the treatment of AD and other tauopathies [89].

Passive immunotherapy targeting tau protein represents a promising disease-modifying strategy for AD. Development of anti-tau mAbs is primarily focused on inhibiting tau aggregation, blocking seeding, and preventing the intercellular spread of pathological tau [90]. Recent studies indicate that primary neuronal tau protein can be released into the extracellular space, and its secretion may contribute to the propagation of tau pathology [91,92]. Therefore, monoclonal antibodies directed against tau protein may limit tau pathology by affecting both its intracellular and extracellular forms.

Neuronal membrane endocytosis may facilitate the uptake of therapeutic antibodies. As an example, the anti-P-tauS422 antibody (MAb86) recognizes membrane-associated P-tauS422, undergoes endocytosis, and is subsequently cleared intracellularly through the lysosomal degradation pathway [93]. Neuronal uptake of the P-tauS422 antibody has been shown to reduce the progression of tau pathology in a mouse model of Alzheimer’s disease [93]. It has been demonstrated that monoclonal antibody (mAb) complexes in the extracellular space can be internalized by neurons through FcγRII/III receptor-mediated, clathrin-dependent endocytosis and subsequently degraded via the endosome–autophagosome–lysosome system [94]. Additionally, in the extracellular space, tau mAb complexes are readily taken up and degraded by murine microglia via Fc receptors and lysosomes [95,96]. Intracellular receptors play a role in mediating the effects of anti-tau monoclonal antibodies. Specifically, TRIM21 is recruited to mAb–tau complexes in neurons and facilitates the neutralization of tau seeds through a proteasome- and valosin-containing protein-dependent degradation pathway [97].

Clinical trials of passive immunotherapy that target tau protein are ongoing for monoclonal antibodies such as ABBV-8E12, gosuranemab, zagotenemab, UCB0107, and semorinemab. These agents are humanized antibodies, belonging to the IgG1 or IgG4 subclasses, whose molecular targets include tau protein in various forms, such as monomers, oligomers, NFTs, or extracellular tau [67,98]. As shown in Table 3, clinical trials of most of the listed anti-tau antibodies have so far failed to achieve the expected therapeutic effects. Despite targeting different molecular forms of tau (monomer, oligomer, aggregates) and various epitopes, none of these antibodies significantly slowed disease progression or reduced tau accumulation in the brain, with the exception of semorinemab in moderate AD, which showed partial slowing of cognitive decline in one of the primary endpoints but had no effect on tau accumulation.

#### 3.3.3. Limitations of Therapeutic Antibodies Approach in Tauopathies

The widespread failure of tau-targeting antibodies in clinical trials is a complex issue, and it highlights several fundamental challenges in targeting tau in AD and related tauopathies. One major problem is the multiple conformations and post-translational modifications of tau. Tau undergoes phosphorylation, truncation, glycation, acetylation, and conformational changes that vary between patients and disease stages [103]. An antibody targeting one specific epitope may miss other pathological forms of tau, allowing neurodegeneration to continue. Disease heterogeneity may cause the single-epitope approach to be insufficient for a broad clinical effect. Another issue is the timeliness of the cure intervention. Many trials enroll patients with mild to moderate Alzheimer’s disease, when the tau pathology is already widespread [104]. Reducing extracellular tau at this stage may not halt the intracellular cascade that has already caused synaptic loss and neuronal death. Many tau antibodies bind monomeric or oligomeric tau, but not necessarily the most toxic species. Like amyloid antibodies, tau antibodies have very low brain penetration [105]. Even if the target is extracellular tau, the effective concentration at the site of the pathology may be too low to meaningfully reduce its pathology.

## 4. The Mechanistic Linking of Aβ and Tau

Extensive evidence supports a pathological interaction between Aβ and tau in Alzheimer’s disease. In mouse models, tau-targeted passive immunization ameliorates cognitive impairments, highlighting the functional relationship and potential synergistic effects of Aβ and tau in disease progression [106]. Simulation modeling in AD patients has consistently demonstrated that tau propagation is accelerated in brain regions exhibiting higher Aβ burden across all Braak stages [107].

At the synaptic level, Aβ oligomers activate extrasynaptic glutamate receptors, particularly N-methyl-D-aspartate receptors (NMDARs), which, in turn, stimulate AMP-activated protein kinase (AMPK). Activated AMPK phosphorylates dendritic tau, promoting its dissociation from microtubules and facilitating tau aggregation [108]. At the proteomic level, Aβ exerts broad effects on tau, TAR DNA-binding protein 43 (TDP-43), and heterogeneous nuclear ribonucleoproteins in human induced pluripotent stem cell-derived neurons [109]. Indirect interactions between Aβ and tau may also be mediated by the APOE4 allele, which can bind directly to Aβ and activate downstream kinases that promote tau phosphorylation [110]. Interactions between Aβ and tau exacerbate mitochondrial impairment and neuronal loss in AD and lead to significant disruptions of neuronal circuitry in experimental mouse models [110,111]. Furthermore, the synergistic toxicity of Aβ and tau leads to hyperactive neuronal signaling and downregulation of synaptic gene expression in TgAPP/PS1 mice with conditional expression of human tau on a tau-null background [112]. In vitro studies further support a bidirectional cross-seeding mechanism in which Aβ and tau mutually promote each other’s aggregation [113]. Consistent with this concept, inhibitors targeting the Aβ core attenuate Aβ-mediated tau pathology and decrease the seeding of both proteins, suggesting the existence of a common epitope that facilitates cross-seeding [114]. Collectively, these findings highlight the direct and indirect mechanisms underlying Aβ–tau synergy and underscore the therapeutic potential of targeting their interaction to inhibit pathological aggregation [Figure 2].

## 5. Medications Targeting Both Tau and Aβ

Drugs that simultaneously target both Aβ and tau are an active but still largely experimental area in AD therapeutics. Most approved drugs still focus on amyloid alone, but there is growing recognition that dual-target approaches may be necessary because Aβ and tau act synergistically in disease progression. Dual Aβ and tau targeting is one of the most promising next-generation strategies. Most of the drug candidates are still at the preclinical or early clinical stages. Examples of dual targeting therapies include BEY-2153 to block or reverse aggregation of both Aβ and tau by inhibition of tau hyperphosphorylation and APP/Aβ pathways [115], AS-603/AS-701 (amyloid solution) for the clearance of both Aβ and tau oligomers/plaques [116], or TREVENTIS (Treventis’s corporation) to inhibit misfolded oligomers of both proteins. There are also physically engineered molecules with dual binding domains, known as bivalent/hybrid compounds, that are still in the early research stages. One of the examples is 2,4 thiazolidinedione-based derivatives, designed to bind both Aβ and tau simultaneously, with established activity in cell and animal models [117]. Furthermore, some dual-pathway modulators targeting shared upstream mechanisms, such as protein misfolding, phosphorylation pathways, and proteostasis/clearance systems, are also considered therapeutic approaches. Both kinase inhibitors and proteostasis enhancers are considered candidates because they can influence both tau and amyloid pathways [118,119].

## 6. Conclusions and Future Perspectives

The multiple manifestations of aberrant AD molecular features, including soluble oligomers, protofibrils, and insoluble fibrillar plaques, suggest that distinct Aβ/tau species may contribute differently to disease pathogenesis. This molecular heterogeneity shows the importance of selectively targeting the most neurotoxic forms of Aβ/tau. Indeed, more recent antibodies have been designed to preferentially bind aggregated or intermediate Aβ/tau forms, which are thought to play a central role in synaptic dysfunction and neuronal injury.

It is not yet known when and how often treatment should be repeated to achieve maximum effectiveness in humans. Therefore, optimizing the timing of preventive measures seems to be a key factor in effective treatment. It is important to evaluate whether strategies that enhance antibody penetration into the brain, such as brain-shuttle-antibody constructs or ultrasound-mediated delivery, could reduce the number of treatments required for effective therapy. As discussed in Section 3.3.3, reducing extracellular tau in the later stages of disease may not be sufficient to prevent the intracellular cascade that has already led to synaptic loss and neuronal death. Many anti-tau antibodies primarily bind monomeric or oligomeric tau but may not effectively target the most neurotoxic species. Similar to anti-Aβ therapies, tau antibodies also exhibit limited brain penetration, which affects their therapeutic efficacy. Although most dual-target strategies remain in preclinical or early clinical stages, approaches that simultaneously target both amyloid-β and tau are increasingly considered as the most promising next-generation therapeutic strategies against AD.

## Figures and Tables

**Table 2:** Overview of passive immunization as a therapy against AD.

Antibody	Target Mechanism	Key Clinical Findings	Status/Notes	References
**Solanezumab**	Soluble monomeric Aβ	Reduced amyloid in the brain (PET imaging); no significant cognitive improvement in large trials	Phase III trials completed; no regulatory approval	[77]
**Crenezumab**	Multiple forms of Aβ (monomer, oligomer)	Safe in early trials; limited evidence of cognitive benefit	Development mostly halted; evaluated in familial AD trials	[78]
**Gantenerumab**	Fibrillar Aβ plaques	Dose-dependent plaque reduction; ongoing studies to evaluate cognitive outcomes	Phase III trials ongoing/partially completed	[78]
**Aducanumab**	Aggregated Aβ (plaques & oligomers)	Robust plaque clearance; modest slowing of cognitive decline in some trials	FDA-approved (2021) via accelerated approval; ARIA monitoring required	[78,79]
**Lecanemab**	Soluble protofibrils of Aβ	Significant plaque reduction; slowing of cognitive decline in early AD	FDA-approved (2023); disease-modifying therapy; ARIA monitoring required	[80]
**Donanemab**	N-terminal pyroglutamate Aβ plaques	Rapid plaque clearance; slowing of cognitive decline in early AD	FDA-approved (2023); disease-modifying therapy; ARIA monitoring required	[81]

**Table 3:** Example of passive immunotherapies targeting tau in clinical trials.

Antibody	Epitope	Target Type	Patients Population	Clinical Autcome	References
Gosuranemab	15–22	Monomer, fibrils, insoluble	Mild AD	Failed to slow cognitive decline; no tau-PET benefit	[99]
Zagotenemab	7–9, 312–322	Aggregates, soluble	Early symptomatic patients of AD	Failed to slow cognitive decline	[100]
Tilavonemab	25–30	Extracellular tau, soluble	Early AD	Failed to slow cognitive decline and reduce tau deposits	[101]
Semorinemab	6–23	Monomeric, oligomeric	Prodromal to mild AD	Failed to slow progression; moderate AD study showed 42% slowing on one endpoint, but no change in tau accumulation	[102]

## Data Availability

No new data were created or analyzed in this study. Data sharing is not applicable to this article.

## Abbreviations

Aβ	β-amyloid
AβPP	β-amyloid precursor protein
AD	Alzheimer’s disease
ApoE	apolipoprotein E gene
ARIA	Amyloid-Related Imaging Abnormalities
AMPK	AMP-activated protein kinase
BBB	Blood–brain barrier
GSK-3	glycogen synthase kinase-3
FDA	U.S. Food and Drug Administration
FTDP-17	frontotemporal dementia and Parkinsonism linked to chromosome 17
mAb	monoclonal antibody
MTs	microtubules
NFTs	neurofibrillary tangles
NMDA	N-methyl-D-aspartate
PET	positron emission tomography
PHF	paired-helical-filament
PSEN1	presenilin 1gene
ROS	reactive oxygen species
